# In a split second: Handwriting pauses in typical and struggling writers

**DOI:** 10.3389/fpsyg.2022.1052264

**Published:** 2023-01-06

**Authors:** Mariona Pascual, Olga Soler, Naymé Salas

**Affiliations:** ^1^Research Center for Psychological Science (CICPSI), Faculdade de Psicologia, Universidade de Lisboa, Lisboa, Portugal; ^2^Facultat de Ciències de l’Educació, Universitat Autònoma de Barcelona, Barcelona, Spain; ^3^Facultat de Psicologia, Universitat Autònoma de Barcelona, Barcelona, Spain

**Keywords:** writing process, writing difficulties, pause analysis, handwriting, letters (alphabet), struggling writers

## Abstract

**Introduction:**

A two-second threshold has been typically used when analyzing the writing processes. However, there is only a weak empirical basis to claim that specific average numbers and durations of pauses may be associated with specific writing processes. We focused on handwriting execution pauses, because immature writers are known to struggle with transcription skills. We aimed to provide an evidence-based account of the average number and duration of handwriting pauses in the mid-Primary grades and to identify process-level markers of writing difficulties.

**Methods:**

Eighty 3rd and 5th graders, with and without writing difficulties, participated in the study. We examined pauses in a handwriting-only task, to be able to isolate those which could only be attributed to handwriting processes. Letter features were considered, as well as children’s handwriting fluency level.

**Results:**

The average duration of handwriting pauses was around 400ms, in line with assumptions that transcription pauses would fall under the 2,000ms threshold. We found that 3rd graders made more and longer pauses than 5th graders. Struggling writers made a similar number of pauses across grades than typically-developing children, although they were significantly longer, even after controlling for the effect of handwriting fluency.

**Discussion:**

Our findings provide an evidence-based account of the duration of handwriting pauses. They also suggest that children need fewer and shorter handwriting pauses as they progress in automatizing transcription. However, some young writers struggle with letter formation even after 3 to 5 years of instruction.

## 1. Introduction

It is a well-established fact that writing development is best understood from a process perspective (Hayes and Flower, 1980; Berninger et al., 1996; Alamargot and Chanquoy, 2001; Graham et al., 2018). Much like speaking, when writing we only produce a number of words at a time, before needing to pause (Chenu et al., 2014). Writing pauses are defined as periods of graphic inactivity, and pausing time can account for up to half the time to compose a text (Strömqvist and Wengelin, 1999; Alamargot et al., 2007). Despite the pervasive presence of pauses during written composition, the reasons why writers of different competence levels pause are not well understood.

Pauses during text production could be the result of immature transcription, such as difficulty to retrieve letter formation motor programs or spelling representations, or of the execution of higher-level writing processes, like planning or revising (Olive et al., 2009; Alamargot et al., 2010). As children’s transcription becomes more fluent overtime, the duration and number of pauses, on average, decreases (Alves and Limpo, 2015; Palmis et al., 2017). This developmental trend points to the pivotal role of transcription skills in the management of the writing process. For this reason, we examined pauses exclusively related to transcription processes; more specifically, to handwriting execution.

### 1.1. Methodological issues in the study of writing pauses

Current research often reports diverging findings with regards to what do pauses reveal about the writing process. For example, it is not clear whether more pauses indicate more or less writing competence. For example, Connelly et al. (2012) reported a negative correlation between the number of pauses and a measure of text quality, whereas a group of undergraduate students in Alamargot et al.’s (2010) study paused more often than a group of 5th graders. In this sense, Ailhaud et al. (2016) found evidence that the activity during pauses may change as a function of the syntactic unit at hand in combination with the age and expertise of participants. They found that pauses before T-units of text were longer for older students (Grade 9), while pauses before highly-governed clause constituents (e.g., subordinate clause) became shorter with age.

The conflicting findings in the literature on writing pauses may be related to the fact that studies differ in how they define pauses. A majority of studies have defined a pause as a period of graphic inactivity of 2,000 ms or longer, which is *assumed* to be devoted to higher-level writing processes (Alves et al., 2019). Pauses under that threshold have been *assumed* to be due to the execution of transcription (i.e., lower level) processes (e.g., handwriting, spelling; Wengelin, 2006; Brizan et al., 2015; Medimorec and Risko, 2017).

However, there are at least two methodological issues with the “default” 2,000 ms threshold and what it entails. First, studies differ widely in their adoption of a pause threshold. A review of studies that collected process data of texts written by hand (Table 1) shows that some have looked at pauses starting below 100 ms (e.g., Chenu et al., 2014; Drijbooms et al., 2020), while others measured pauses at or above 10,000 ms (Prunty et al., 2016a,b). Therefore, there is great disparity in the very definition of what a pause is and at which point may pauses be attributed to the execution of transcription or high-level writing processes.

**Table 1:** Summary of tools, thresholds and tasks in a selection of previous studies.

	Tool	Age range	Threshold	Task
Alamargot et al., 2010	Eye and pen	12 to 18 yo and adults	15 ms	Narrative text
Alamargot et al., 2021	Eye and pen	10 to 12 yo	20 ms	Name writing Alphabet task
Alves and Limpo, 2015	Handspy	7 to 13 yo.	2000 ms	Narrative text Opinion essay
Alves et al., 2016	Handspy	7 and 8 yo.	30 and 2000 ms	Narrative text Handwriting tasks
Alves et al., 2019	Handspy	7 and 8 yo.	30 and 2000 ms	Narrative Text Handwriting task
Beers et al., 2017	Inputlog Eye and Pen	9 to 15 yo.	2000 ms	Narrative Text Handwriting task Copy Task
Chenu et al., 2014	Eye and Pen	10 to 15 yo.	15 ms	Narrative text Expository text
Connelly et al., 2012	Eye and Pen	11 yo.	2000 ms	Writing task “One day I had the best weekend ever..”
Dockrell et al., 2019	Eye and Pen	8 and 11 yo.	2000 ms	CBM Writing Task (McMaster and Espin, 2007)
Drijbooms et al., 2020	Eye and Pen	11 yo and adults	35 ms	Narrative text
Maggio et al., 2012	Eye and Pen	7 to 15 yo.	–	Narrative Text
Olive et al., 2009	Eye and Pen	undergraduates	>250 ms (writing) < 250 ms (copy)	Narrative and copy task
Prunty and Barnett, 2017	Eye and Pen	8 to 14 yo.	30 ms	DASH (Barnett et al., 2007)
Prunty et al., 2014	Eye and Pen	8 to 14 yo.	30 ms	Free writing task (DASH; Barnett et al., 2007)
Prunty et al., 2016a	Eye and Pen	8 to 14 yo.	10,000 ms	Free writing task (DASH; Barnett et al., 2007)
Prunty et al., 2016b	Eye and Pen	8 to 14 yo.	10,000 ms	Free writing task (DASH; Barnett et al., 2007)
Prunty et al., 2020	Eye and Pen	8 to 14 yo.	–	DASH (Barnett et al., 2007)
Van Hell et al., 2008	OASIS	10 to 25 yo.	–	Narrative and expository tasks

Second, the vast majority of investigations examined pauses taking place during text composition, and made an assumption about what underlay different pause durations, instead of adopting a data-driven approach. For example, Prunty et al. (2014) defined four types of pauses, based on their duration, which were based on previous literature: (1) between 30 to 250 ms, which were assumed to indicate letter-formation processes; (2) between 250 ms to 2 s, considered to indicate between-letter pauses; (3) between two to 4 s, signaling word-level pauses; and (4) above 4 s, which were considered to represent a higher level writing process (generating ideas) or to indicate “fatigue or a lack of writing ideas” (p. 2898). Notably, the pause duration range for handwriting execution was based on a study with only five participants, including school children and up to expert adult writers (Alamargot et al., 2010). Similarly, the threshold assumed for word-level pauses (250 ms–2 s.) was adapted from findings for Hebrew-speaking children with or without a coordination disorder (Rosenblum and Livneh-Zirinski, 2008). In short, there is only a weak empirical basis to support assumptions about which processes are being executed during pauses. Because most investigations analyzed pauses within the context of a text writing task, it is virtually impossible to safely attribute pauses to one type of writing process (Olive et al., 2009).

### 1.2. Previous research on handwriting pauses

To understand the duration of handwriting pauses, some studies have measured the length of inter-letter or inter-syllable intervals during handwriting execution. The values obtained in these studies can be used as a guideline for establishing the thresholds for handwriting pauses. Studies on syllable programming by preschool children found inter-letter and inter-syllable intervals of a minimum of 1 second (Soler-Vilageliu and Kandel, 2012), whereas a study with adults comparing inter-letter intervals in simple and complex syllabic structures reported average values of around 90 ms in simple syllables and of up to 155 ms for complex syllables (Kandel et al., 2008). All in all, this body of evidence seems to indicate that pauses at or around 100 ms are related to the motor execution of handwriting in adults, but children may require considerably longer pauses to deal with tracing letters and spelling out words. In this study we identified all handwriting pauses starting at 100 ms. A minimum of 100 ms has been found to be the lowest stroke-duration threshold for the analysis of writing processes (Maarse et al., 1989; Plamondon, 1991; Schomaker, 1991; Van Galen, 1991; Kandel and Soler, 2010; Kandel and Perret, 2015).

In addition, some properties of letters are likely to influence the nature of handwriting pauses. Letter case (i.e., uppercase v. lowercase) and letter type (e.g., cursive v. print) are relevant factors that should be considered when studying written execution. Different allographs may pose different cognitive and motor demands, especially to young writers (see Pagliarini et al., 2015 for a study where case was controlled across populations), but also adult writers seem to be affected by the type of letter they write. For example, Bourdin and Fayol (1994) showed that when adults were told to remember a series of words using uppercase, cursive letters they recalled significantly fewer words than when they used a more familiar script. Finally, familiarity with a particular script type and case may also be influenced by other factors. Teachers in Spain provide instruction on uppercase print writing to children in preschool but, from grade 1 onwards, children are taught to write using cursive letters almost exclusively. Moreover, children’s books are often written in cursive, and after an initial exposure to print uppercase, schools include cursive teaching in their curricula (Graham et al., 1998; Schwellnus et al., 2012; Bara et al., 2016).

In order to contribute to clarifying these conflicting findings, in this paper we investigated the nature of a specific type of pause: Those due exclusively to handwriting execution. We considered the *alphabet task* (Berninger et al., 1992), given that it taps directly into the retrieval of graphemes from memory and into the visuo-motor integration skills required to reproduce letters in writing (Kim et al., 2011; Arfé et al., 2020). Moreover, this task has been found to efficiently identify handwriting difficulties (Rosenblum and Livneh-Zirinski, 2008). Other types of tasks, such as copying tasks, do not tap into the graphemic buffer, which is intrinsic to any handwriting task (Alstad et al., 2015). As it happens, neuropsychological research has found that letter writing can activate more neural systems than copying or passively recognizing letters, which do not require recruiting as many neural areas (James and Gauthier, 2006). We focused on this type of pause because handwriting has been found to be one of the most constraining processes in early writing development (Alves and Limpo, 2015). In addition, some studies have suggested that struggling writers show an alteration in the nature of their pause patterns (Sumner et al., 2013). Therefore, investigating the nature of handwriting processes should allow us to better understand key drivers of development of writing and potential causes of writing difficulties.

While there is a growing body of evidence suggesting that pauses are sensitive to the expertise of the writer (although it is not clear exactly how so, as pointed above), less is known about process-level markers of writing difficulties. Some studies looking at writing pauses in children with sustained writing difficulties found no differences with a group of typically-developing peers in their average pause time or number (Pascual, 2020). However, other studies have found that dyslexic children pause more often (Sumner et al., 2013, 2014; Beers et al., 2017, 2018) and that there is a rhythmic constraint in children with dyslexia and dysgraphia that hinders their speed which, in turn, has a relationship with reading and language development (Lam et al., 2011; Pagliarini et al., 2015). Alamargot et al. (2021) found that dyslexic children needed more time to produce letters and made more handwriting pauses (especially those under 200 ms) than age-matched peers. However, they did not consider pauses above 1,000 ms duration; thus, it is not clear whether there could be longer pauses during handwriting execution. In this paper, we investigated whether a group of struggling writers showed differences in the nature of handwriting pauses in comparison with a group of age-matched, typically-developing students.

### 1.3. This study

The present study aimed to provide an evidence-based account of the average number and duration of handwriting pauses in a language with a semi-consistent orthography (Catalan). Four main characteristics of the study make it an improvement over previous research on writing pauses: (1) it examined pauses in a handwriting-only task (writing the alphabet letters continuously for a short period of time). In this way, we were able to isolate pauses which could only be attributed to handwriting processes and not to other lower- or higher-level writing processes, in contrast to most previous studies; (2) it considered all pauses starting at 100 ms, and controlled for key properties of letters (e.g., type of script, number of strokes per letter); (3) it tested the effect of Grade (3rd, 5th) and Population (typically-developing v. struggling writers), thus allowing to observe any developmental trends and to identify process-level markers of writing difficulties; and (4) used a friendlier longhand writing device (smart pen), rather than other, less ecological alternatives, such as a writing tablet or a keyboarding setting.

Our research questions were as follows,

1. What is the average duration and number of pauses that are due to handwriting execution?
2. Which letter features (i.e., number of traces required, cursive v. print) influence the average number and duration of handwriting pauses?
3. Do pause duration and number decrease as a function of schooling experience?
4. Is pause duration or number of pauses a process-level marker of writing difficulties?

With regards to research question (1), we hypothesized that handwriting pauses would have an average duration under 2,000 ms, in line with suggestions from previous research (Alves et al., 2007; Wengelin, 2007; Prunty et al., 2014). With regards to research question (2), we hypothesized that letters involving more strokes and letters written in print would be associated with longer pause durations, given that they increase the difficulty of tracing the letter, while the cursive-linking features entail fewer pen lifts (Morin et al., 2012). Our hypothesis for research question (3) was that 5th grade children would show shorter pause durations, and fewer pauses per letter on average, than 3rd grade children, as the former would have more automatized transcription processes than the latter (Alves and Limpo, 2015). Finally, with regards to research question (4) we expected struggling writers to have slower handwriting fluency, which would be reflected in longer average pause durations and more pauses per letter in comparison to typically-developing peers (Sumner et al., 2013).

## 2. Materials and methods

### 2.1. Participants

**Figure 1:**
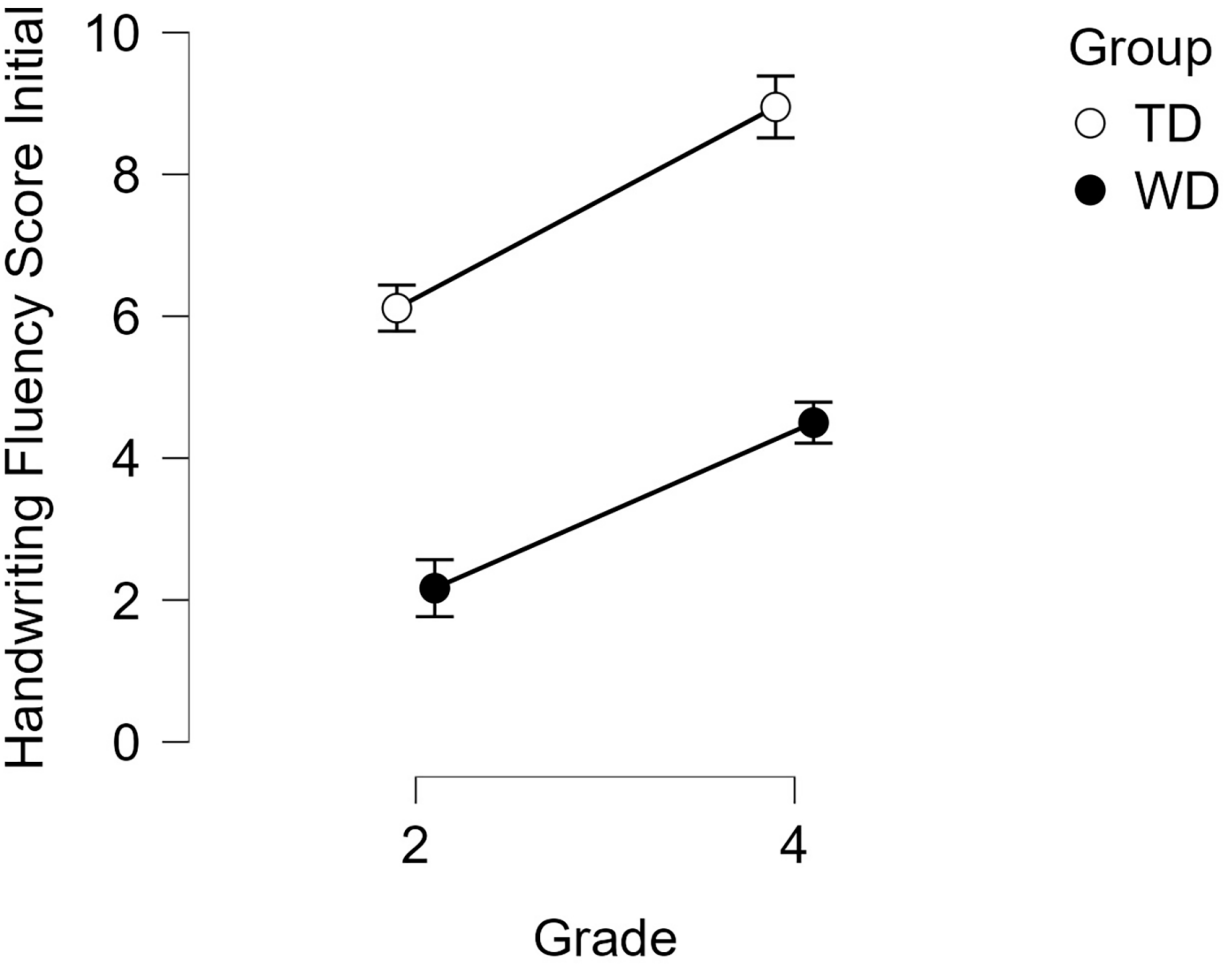
Descriptive plots of the initial identification handwriting task, as found in Pascual (2020).

Drawing from a larger study on literacy development, we randomly selected a group of 83 students, of which 67 were typically-developing (TD) students (34 boys) attending 3rd and 5th grade (Table 2 shows participants’ distribution and demographic characteristics), and 16 students (12 boys) struggled with handwriting. The families of all participants consented to their participation in the study. Children with handwriting difficulties (HWD) had been identified in a previous study by their consistent performance below -1SD (*p* < 0.001) across two handwriting tasks (Figure 1 shows initial differences per group and grade in the alphabet task; Pascual and Salas, 2021). This initial identification, carried out when children were attending grades two or four, was further supported by a follow-up study 16 months later, where we found that their poor performance in handwriting tasks persisted, and it affected several text-based measures, including text generation and spelling (Pascual, 2020). Of the initial sample, three students were discarded for writing letters as strings of cursive without lifting the pen between them as indicated by the administrator. Thus, our final sample of TD students was of 64 participants (33 boys).

**Table 2:** Demographic data and characteristics of participants across groups.

	TD		WD	
	Grade 3 *M* (SD)	Grade 5 *M* (SD)	Grade 3 *M* (SD)	Grade 5 *M* (SD)
*N* (boys)	30 (14)	34 (19)	10 (8)	6 (4)
Mean age	8;8 (0;4)	10;10 (0;3)	8;8 (0;3)	10;10 (0;4)
HWF* Identification Score	6.11 (1.92)	8.95 (2.75)	2.45 (0.82)	4.43 (0.54)
ISEI Score	59.35(14.35)	65.39 (6.28)	58.86 (16.76)	60.38 (15.02)

TD = typically developing group; WD = writing difficulties group; HWF = handwriting fluency (alphabet task); ISEI = International Socio-economic Index Score (Ganzeboom et al.,1992).

* The data was collected when struggling students were identified a year before the current study; that is, when the Grade 3 group was attending 2nd grade, and the Grade 5 children were attending 4th grade.

Given the high variability in students’ proficiency with Catalan, which is both the language of instruction but also a minority language, it was important to ensure that our participants belonged to a context in which Catalan was frequently used, not just within schools, but particularly outside the school setting. Therefore, students were recruited from three public primary schools in medium-high socioeconomic districts in Barcelona, a bilingual Catalan-Spanish community in Spain. Usage of Catalan is linked to skilled occupation and to higher academic achievement in adults (Idescat, 2015). Moreover, we administered sociolinguistic questionnaires to assess the Socio-Economic Status, using Ganzeboom et al. (1992) International Socio-Economic Index (ISEI). The average ISEI score was 62.5 (SD = 11.34); therefore, on average, these students’ families ranked in the 70th percentile of the ISEI (a detailed ISEI score per group and grade can be found in Table 2). No significant statistical differences were found between groups on ISEI score, *F* (2, 70) = 0.807, *p* = 0.372.

### 2.2. Tasks and measures

**Alphabet task**. Students had 60 s to write the alphabet as many times and as they could and as quickly as possible. To obtain an estimate of *handwriting fluency*, we counted the number of well-written letters within the first 15 s (after Berninger et al., 1992). To explore pause duration and pause number, we identified all pauses starting at 100 ms. Each pause log was assigned to the corresponding letter to be able to compute the *number of pauses per letter*, and we also recorded each *pause duration* (in milliseconds). Reliability (ICC) between the first author and an RA on 20% of the handwriting tasks was.884.

In addition, we considered some letter properties. Although children had been instructed to use lowercase print letters, they did not always comply. Therefore, each letter production was coded for script type, distinguishing between cursive, print lowercase, or print uppercase, and this information was used in all analyses. In addition, the number of strokes needed to trace each letter (based on Soler and Kandel, 2009, who adapted Meulenbroek and Van Galen, 1990) was used as an independent variable in all analyses.^1^

### 2.3. Procedure

The task was part of a larger test battery administered at the end of the school year (May–June) of 3rd and 5th grade in two sessions. Students were assessed in groups of five in a quiet place at the school across two 40-min sessions. The handwriting task was carried out last in session 1, preceded by an opinion essay writing task and a spelling task.

The handwriting task was carried out using Neo Smartpens M1®, writing on Neonotes Pads®. Smartpens connect through infra-red cameras to record the coordinates of the writing strokes. M1 pens weigh 17.4 g and are 149.6 mm long, being only slightly heavier than a regular pen. The sheets on N idea pads® are ruled line sheets with micro-dotted patterns (NcodeTM technology), which allow pens to record the coordinates of the stroke, recognizing the spatial–temporal traces on a specific page. This recording is then paired to the Neonotes® app, from which data is transferred *via* Bluetooth to a tablet.

### 2.4. Data analyses

The output from the Neonotes app was analysed using Handspy®, a software specifically developed to assess online writing processes (De Sousa and Leal, 2013; Monteiro and Leal, 2013; Alves, 2019). Each page is transformed through the XML markup language to a protocol, which displays the strokes and pauses in different logs. Pause duration is automatically displayed, as well as stroke duration. Before analyzing the measures, protocols were cleaned to account only for the pause duration and pause number within the task and thus eliminate registries that did not reflect task execution time (e.g., writing down the name, pre-task pausing time, post-task pausing time).

We ran cross-classified multilevel models (CCMLM) in MPlus version 8 (Muthén and Muthén, 2017). CCMLM allowed a thorough analysis of within-letter pausing data, as well as between-letter and between-student differences (Van den Noortgate et al., 2003; Fielding and Goldstein, 2006). This type of modelling has been already found useful in previous studies that conducted letter-feature analyses across both letters and students (e.g., Kim et al., 2010; Piasta and Wagner, 2010), due to its ability to consider “participants as raters of similar items” (Kim et al., 2010, p. 315), which in this case are letters, nesting pause duration and number not only within letters but also within participants. In other words, CCMLM enables within-item, between-item, and between-participant level interaction, controlling for each coefficient’s standard error (Shi et al., 2010).

## 3. Results

Descriptive statistics of pause duration and pause number, as well as the handwriting fluency score, by group and grade, are reported in Table 3. In grade 3, pauses lasted for 414 ms on average for the typically developing group (SD = 360 ms). For the WD group, average pause duration was 536 ms (SD = 302 ms). Pausing rate was around 1.8 pauses per letter for both groups. In grade 5, handwriting pauses lasted around 360 ms across groups, and 1.42 pauses were made, on average, for each letter, also in both the TD and the WD groups.

**Table 3:** Descriptive statistics of HWF score, pause number and duration by group and grade.

	TD		WD	
	Grade 3 *M* (SD)	Grade 5 *M* (SD)	Grade 3 *M* (SD)	Grade 5 *M* (SD)
HWF score	11.99 (3.65)	17.61 (7.17)	8.6 (4.49)	11.9 (3.07)
Mean pause duration per letter (ms)	414.15 ms (359.97 ms)	358.52 ms (282.37 ms)	536.11 ms (301.98 ms)	360.68 ms (301.98 ms)
Mean pause number per letter	1.84 (0.90)	1.43 (0.67)	1.7 (0.83)	1.42 (0.68)

TD = typically developing group; WD = writing difficulties group; HWF = Handwriting Fluency Score (no. of letters written).

Our CCMLM models used Bayesian (MCMC) estimation (1,000 iterations) to assess both pause duration and pause number considering two levels. The variables included in each level are depicted in a multilevel diagram in Table 4 (following Monsalves et al., 2020). At level 1, which tested variance *within letters*, we estimated a within-letter pause latent factor, a within-letter strokes latent factor, and two within-letter script-type latent factors which included a dummy coding of the possible script types (the latent factor Script1 reflected the contrast between cursive and both low and uppercase print; the Script2 latent factor reflected the contrast between lowercase print and both cursive and uppercase print). All latent factors were pseudo-factors, such that factor loadings were fixed to one. This level of analysis allowed us to determine which letter characteristics affected pause duration or number during letter formation. The strokes and the script-type latent factors were regressed on the within-letter pause factor.

**Table 4:** Multilevel diagram in table format.

Level	Level cluster	Variables in pause number	Variables in pause duration
1	Within same-letter level	Pause Number	Pause duration within letter
		Strokes per letter	Strokes per letter
		Case Contrast (1)	Case Contrast (1)
		Case Contrast (2)	Case Contrast (2)
2-a	Between different-letter level	Pause number per letter	Pause duration per letter
		Strokes per letter	Strokes per letter
		Case Contrast (1)	Case Contrast (1)
		Case Contrast (2)	Case Contrast (2)
2-b	Between-subject level	Pause number per letter per subject	Pause duration per letter per subject
		Grade	Grade
		Group	Group
		Score in Hw Fluency	Score in Hw Fluency

At level 2 we distinguished two sub-levels to account for differences between letters (level 2a) and between students (level 2b). Similarly to level 1 above, in the between-letters level, we estimated a latent factor of the pause duration/number per letter, another latent factor of the number of strokes per letter, and latent factors for the two dummy variables reflecting contrasts between script types. All latent factors reflected the actual observed value. By creating pseudo-latent factors, we could run analyses across levels. In the between-subjects sublevel (2b), we estimated a latent factor of pause duration/number, which was fixed to 1. This factor was regressed on Grade, the handwriting fluency score, and on Group (that is, whether students had writing difficulties or not). Final model results can be found in Table 5.

**Table 5:** Pause duration and pause number cross classified multilevel model per levels and the proportion of variance explained by the regression per level (R^2^).

*Estimate*		*Post. S.D*	*p*	*95% C.I*		R^2^ [95% CI]
				*L 2,5%*	*U 2,5%*	
Within-letters level variables						
Pause Duration within letters						0.24 [0.10–0.41]
Strokes	−0.038	0.164	0.496	−0.240	0.252	
Script1	0.191	0.171	0.213	−0.220	0.331	
Script2	0.092	0.108	0.048	−0.010	0.320	
Pause Number within letters						0.44 [0.11–0.75]
Strokes	0.227	0.514	0.275	−0.414	1.467	
Script1	−0.837	0.566	0.000*	−1.828	−0.127	
Script2	−0.500	0.197	0.013*	−0.771	−0.097	
Between-letters level variables						
Pause duration between letters						0.44 [0.04–0.97]
Strokes	−0.008	0.074	0.447	−0.154	0.154	
Script1	0.015	0.113	0.447	−0.266	0.235	
Script2	0.004	0.100	0.489	−0.222	0.200	
Pause number between letters						0.54 [0.08–0.91]
Strokes	0.036	0.416	0.453	−0.832	0.881	
Script1	−0.154	0.670	0.414	−1.256	1.276	
Script2	0.219	0.451	0.314	−0.634	1.161	
Between subjects level variables						
Pause duration between subjects						0.74 [0.48–1.00]
Grade	−0.026	0.021	0.089	−0.069	0.016	
Group	0.006	0.027	0.414	−0.040	0.065	
Score in HWF	−0.012	0.003	0.000*	−0.017	−0.006	
Pause number between subjects						0.78 [0.54–0.97]
Grade	−0.133	0.056	0.017*	−0.238	−0.009	
Group	−0.071	0.067	0.115	−0.212	0.056	
Score in HWF	−0.033	0.005	0.000*	−0.045	−0.025	

CI = confidence interval; L = lower; U = upper; Script1 = contrast between cursive vs. lower and upper case; Script2 = contrast between lower case vs. cursive and upper case; HWF = handwriting fluency (Alphabet task). *p < 0.05.

Before conducting the final analyses we identified outliers at each level of analysis and for each dependent variable. For the pause duration analyses, at level 1 we excluded pauses that were 3 SDs above the mean (i.e., ≥ 2.030 ms), resulting in a loss of 92 data points (0.02% of data). Of these data, 42% was from WD children, and 58% was from TD children. At level 2a we excluded 8 instances of children writing the letter “ñ,” which does not belong to the Catalan alphabet (but to the Spanish one); therefore, at this level the cluster was reduced from 27 to 26 items. At level 2b one child was excluded due to her performance in the handwriting fluency task, which was well outside normalcy parameters (i.e., > 22 letters within the first 15 s of the task or 5.7 SDs). Therefore, 92 data points were removed, and the size of the 2b level cluster went from 80 subjects to 79. Overall the total loss of data was 3.31% of observations (from 5,793 pauses logged to 5,601 pauses).

In the pause number analysis, at level 1, 120 data points were excluded because the number of pauses within-letter was above 3 SDs (i.e., letter executions which required four or more pauses per letter were excluded). This corresponded to 3.40% of all letter executions (3,528). Because we had included a letter-ID index variable, identifying the 26 letters of the Catalan alphabet, no data were excluded at level 2a. Last, at level 2b we excluded the same outstanding student due to her handwriting fluency score (cluster size = 79). Unless otherwise specified, we report unstandardized coefficients.

### 3.1. Pause duration

Results showed that, at the within-letter level, pause duration was not significantly explained by the number of strokes, b = −0.04 (PSD = 0.16), *p* = 0.496, or script (i.e., lowercase print, uppercase print, or cursive), b = 0.19 (PSD = 0.17), *p* = 0.213, for the contrast between cursive and both low and upper-case print, and b = 0.09 (PSD = 0.11), *p* = 0.048, for the contrast between lowercase print and both cursive and uppercase print. At the between-letter level, the number of strokes did not correlate significantly with within-letter pause duration, b = −0.01 (PSD = 0.07), *p* = 0.447. Script type did not have a significant effect either, on the average between-letter duration, b = 0.02 (PSD = 0.11), *p* = 0.447, for the cursive v. print contrast; and b = 0.004 (PSD = 0.10), *p* = 0.489, for the lowercase print v. cursive + upper-case print contrast.

At the between-student level, Grade did not have a significant impact on the average pause duration, b = −0.03 (PSD = 0.02), *p* = 0.089. Moreover, children with writing difficulties tended to make pauses that were similar in length to typically-developing students, b = 0.01 (PSD = 0.03), *p* = 0.414. In contrast, students’ handwriting fluency score did influence pause duration significantly, b = −0.12 (PSD = 0.003), *p* < 0.001, such that more fluent handwriting was associated with overall shorter pauses. Notably, the correlations between the handwriting fluency score with Grade and with Group were *r* = 0.55 and *r* = −0.45, respectively. This, in addition to the fact that the handwriting fluency score is on a larger scale, in comparison with both Grade and Writing-difficulty status, which are binary variables, could be suppressing the “real” effect of our variable of interest. Therefore, we reran the analysis, excluding the handwriting fluency variable. This analysis showed that both Grade and Group did have a significant impact on average pause duration, b = −0.08 (PSD = 0.02), *p* < 0.05 (Grade); b = 0.06 (PSD = 0.02), *p* < 0.05 (Group): younger children and children with writing difficulties made longer pauses, on average. The model explained a considerable proportion of the variance at the between-student level, *R*^2^ = 0.795 (PSD = 0.12), *p* < 0.001.

### 3.2. Pause number

At the within-letter level, the average number of pauses was not significantly explained by the number of letter strokes, b = 0.23 (PSD = 0.51), *p* = 0.275. However, letter type did have a significant effect on the average number of pauses within letters. Children who wrote letters using cursive made overall fewer pauses than those who used print, b = −0.84 (PSD = 0.57), *p* < 0.001. Similarly, children who wrote using lowercase print made overall fewer pauses than children who used either cursive or uppercase print, b = −0.50 (PSD = 0.20), *p* = 0.013. At the between-letter level, neither the number of strokes, b = 0.04 (PSD = 0.42), *p* = 0.453, or the script type, b = −0.15 (PSD = 0.67), *p* = 0.414 (cursive v. print), b = 0.22 (PSD = 0.45), *p* = 0.314 (lowercase print v. cursive and uppercase print), significantly affected the number of pauses between letters. Finally, at the between-student level, Grade had a significant impact on the average number of pauses, b = − 0.13 (PSD = 0.06), *p* = 0.017, such that younger children made more pauses per letter, on average. Students’ handwriting fluency score also influenced the average pause number significantly, b = −0.03 (PSD = 0.01), *p* < 0.001, such that more fluent handwriting was associated with overall fewer pauses. However, Group did not have a significant effect on the average number of pauses per letter, b = −0.07 (PSD = 0.07), *p* = 0.115, indicating that children with writing difficulties made a similar number of pauses per letter, on average, as typically-developing students. As with the pause duration results, we reran the analysis excluding the handwriting fluency score, to determine whether this variable was suppressing an effect of Group on the number of pauses. Our second analysis indicated that this was not the case, as Group was not a significant predictor of number of pauses either, b = 0.07 (PSD = 0.05), *p* > 0.05, while the effect of Grade remained significant also in this second analysis, b = −0.27 (PSD = 0.03), *p* < 0.05. The model explained a considerable proportion of the variance at the between-student level, *R*^2^ = 0.728 (PSD = 0.13), *p* < 0.001.

## 4. Discussion

This study aimed to examine to identify and characterize the nature of pauses that are due to handwriting processes during a handwriting task. Moreover, we aimed to ascertain the potential effect of a series of letter properties (e.g., number of strokes required, letter type) on the duration and number of handwriting pauses. Finally, we intended to understand how schooling affects these pauses and whether pause number and/or pause duration could be a marker of writing difficulties. For these purposes, we collected handwritten productions and analysed them thoroughly, identifying within and between-letter factors. Critical to the study design was that pauses should be safely attributed to handwriting execution and not to other writing processes, such as spelling, planning, or revising.

### 4.1. The nature of handwriting pauses in mid-primary

We provided sound evidence that the overall average duration of a handwriting pause, at least in the mid-primary grades in a semi-consistent orthography, is around 400 ms. Our methodological design allowed us to safely attribute pauses to handwriting execution of letters, given that it was the only process required by the task. This is a major change over most previous research on the topic, which has, for the most part, made assumptions about the high-level processes underlying pauses during writing and had overlooked establishing a data-driven threshold for transcription pauses. If transcription processes are considered as moderators or predictors of writing quality (Limpo and Alves, 2017), we have found the need to also account and better define transcription pauses, which will be crucial for understanding early writers text’s composition.

In this sense, beyond a within-automatization task pause duration, transcription pauses may undergo significant changes depending on the task at hand. The cognitive effort that has to be devoted to composing texts changes between genres and schooling level (Olive et al., 2009; Beauvais et al., 2011), and handwritten automatisation plays a determinant role in the amount of cognitive resources available to devote to higher-level writing processes such as organizing ideas, translating ideas into language, or revising (McCutchen, 2000). Studies that explore within-text pause should consider the idiosyncrasies of the task at hand, given that speed, pause duration and increase or decrease of pausing patterns are linked to syntactic constructs (clauses, sentences, paragraphs; van Hell et al., 2008; Ailhaud et al., 2016).

Our finding about the average duration of handwriting pauses conflicts with previous research in English with 9-year-old students, which reported an average pause duration during the alphabet writing task of 722.25 ms (SD = 261.33 ms; Sumner et al., 2013, p. 998). However, it has been suggested that orthographic depth may have an impact on handwriting execution (Kandel and Soler, 2010). All in all, however, our findings and those of Sumner et al.’s (2013) should be reassuring to researchers who considered that transcription pauses were on the shorter end of the continuum, in comparison with other writing pauses, such as planning (e.g., Alves et al., 2008; Olive et al., 2009). Conversely, some studies that investigated handwriting execution pauses specifically may have underestimated their average duration. For example, Prunty et al. (2014) used literature to classify “within-letter” pauses as those occurring under 250 ms, while “between-letter” pauses were estimated to occur within a 250–2,000 ms range. The present paper warns against these types of assumptions, often based on measurements that cannot be unmistakably attributed to a specific writing process. Future studies should attempt to isolate spelling-related pauses, in order to ascertain the pause-duration range for transcription processes.

As for the average number of pauses per letter, our study showed that children made about 1.5 pauses per letter, when they performed the alphabet task. Contrary to pause duration, there are no widely used thresholds of what a reasonable or unreasonable number of pauses should be, or whether certain processes are associated with a higher or smaller number of pauses. However, our findings are relatively well aligned with those reported recently by Alamargot et al. (2021), who investigated handwriting execution pauses in dyslexic French-speaking children, and compared them to groups of age- and spelling-skill-matched controls. The authors obtained pauses using a name+surname writing task, and an alphabet-writing task, thus focusing solely on handwriting execution. Pauses were divided into five categories: P1 = 20–199 ms; P2 = 200–399 ms; P3 = 400–599 ms; P4 = 600–799 ms; and P5 = 800–1,000 ms (Alamargot et al., 2021, p. 168). If we consider the average number of pauses reported across categories, typically-developing, 11-year-old children made about 1.6 pauses per letter in the alphabet task, while both control groups made significantly more pauses of the shortest kind, but did not differ in the rate at which they made other types of pauses. Importantly, the shortest pause type (under 200 ms) was, by far, the most frequent across groups and tasks, suggesting that handwriting execution may be exerting demands at a very low level, which appears to be impaired for some groups of children. One could argue that the children in our study should be making more handwriting pauses, on average, given that they are slightly younger than the age-matched TD children in Alamargot et al.’s (2021) study. However, note that they (1) started counting pauses at 20 ms (while we did so at 100 ms); and (2) are users of French, a considerably more inconsistent orthography (e.g., Caravolas et al., 2020). In this sense, we have already mentioned the possibility that orthographic depth or consistency levels might impact handwriting execution (Kandel and Soler, 2010). Minor nuances aside, our study has contributed to narrowing down the range in the number of pauses that mid-primary children require for handwriting in a semi-consistent orthography (Catalan).

### 4.2. Letter features that affect handwriting execution

The present study was innovative in considering the effect of key letter features, such as the number of strokes required per letter (based on Soler and Kandel, 2009, who adapted Meulenbroek and Van Galen, 1990), as well as the type of script used by children during the task, despite the fact that they were all instructed to use lowercase print letters. Contrary to our expectations, pause duration was unaffected by either letter feature, while the average number of pauses by letter differed as a function of the type of script used by children: Cursive letters required fewer pauses than lowercase print letters, and these, in turn, fewer than uppercase print letters. Cursive script is composed by ‘joined-up letters, continuous movement and few pen lifts’ (Bara et al., 2016, p.89). An interesting educational implication may be derived, then, from these findings, such that handwriting fluency could be facilitated, to some extent (i.e., requiring fewer pauses), by using cursive script. Nevertheless, more research is needed to determine what other facets of handwriting fluency –a crucial factor in the development of writing and tightly associated to writing quality and productivity throughout most of obligatory education (e.g., Graham et al., 1997; Salas and Silvente, 2020) – are impacted by these and other letter features.

### 4.3. Schooling effects on handwriting pauses

Another aim of the study was to determine if handwriting pause duration and number would be influenced by children’s schooling level. We hypothesized that 5th grade children would show shorter pause durations and fewer pauses per letter, on average, than the 3rd grade children, as the former would have more automatized transcription processes than the latter (Alves and Limpo, 2015). As expected, schooling level was significantly involved in explaining individual differences in pause number and duration between students. This was not surprising, given the abundant evidence that handwriting abilities are key factors in learning to write and pose major constraints to the development of writing (e.g., Kim et al., 2011, 2014; Wagner et al., 2011; Dockrell et al., 2019). Handwriting requires a substantial amount of writing resources to young and struggling writers, as it has been reported in investigations that explored written products (Berninger et al., 1992; Bourke et al., 2014; Kim et al., 2014; Kandel and Perret, 2015; Kim and Schatschneider, 2017) and processes (Connelly et al., 2012; Alves et al., 2016, 2018; Beers et al., 2017, 2018; Medimorec and Risko, 2017; Limpo and Alves, 2017). Therefore, the reduction in pause duration and number at the letter level may indicate a developmental integration of transcription skills.

Although the schooling effect on handwriting pauses was clear, children’s score in the alphabet task was the main factor that predicted the average duration of handwriting pauses between subjects, while schooling level did not explain unique variance above and beyond this measure. Only when handwriting fluency was removed from the model did schooling level become a significant factor. This was not the case for the average number of pauses, where schooling level contributed to explaining significant between-subject variance even after controlling for the powerful effect of handwriting fluency scores. We have mentioned elsewhere that the handwriting fluency score provides a larger scale that facilitates establishing a pattern between this skill and pause number or duration, in contrast with the binary variable of Grade. Besides this observation, which is of an operational nature, perhaps a more important conclusion would be that handwriting fluency appears to be a sensitive-enough indicator of handwriting skill, rather than the more sophisticated (and much more difficult to obtain) measures of handwriting pause duration and number.

### 4.4. Handwriting pauses as markers of writing difficulties

It has long been established that writing is best understood as a cognitive process (Hayes and Flower, 1980; Alamargot and Chanquoy, 2001). Longitudinal studies have demonstrated that there are causal effects between different components of writing (e.g., Vilageliu et al., 2016). Intervention studies have repeatedly demonstrated that providing procedural scaffolding to write improves writing competence (e.g., Graham et al., 2012, 2018). More specifically, providing handwriting strategies, from scaffolded learning to remedial instruction (see Alves et al., 2018; Limpo and Graham, 2020 for detailed reviews and examples of best-practices in handwriting teaching), leads to improvements in text quality (e.g., Santangelo and Graham, 2016). Therefore, given that writing processes in general, and handwriting in particular, are causally linked to writing development, we reasoned that features of handwriting execution, such as pauses, could be markers of writing difficulties. For this purpose, we examined whether either handwriting pause duration or number could be process-specific indicators of writing difficulties. Our results showed that children with writing difficulties, identified as having handwriting difficulties (Pascual and Salas, 2021), paused for longer during handwriting execution. However, they made a similar number of pauses per letter as their TD peers. In other words, during handwriting execution, struggling writers do not pause more often, but when they do so, it takes them longer than TD controls to resume handwriting. The finding that struggling writers pause more than their TD peers during handwriting execution is in line with previous studies with dyslexic children and with children matched to dyslexic peers on spelling skills (Sumner et al., 2013). Thus, it would appear that difficulties with handwriting processes could be at the root of struggling writers’ problems to compose text. Similarly, to the Grade effects reported above, though, a simple measure of handwriting fluency was a more important predictor of average pause duration and it needed to be removed for writing status (TD v. WD) to become a significant factor. Again, as with Grade, a non-binary variable would facilitate the detection of a correlational pattern with the outcome variable, rather than a binary one. However, even if handwriting fluency is sufficiently efficacious to identify handwriting difficulties, from a theoretical and a clinical standpoint, it is paramount to understand the mechanisms of these difficulties to arrive at effective remediation strategies. Future research should strive to expand the current findings to build a more complete explanatory picture of handwriting difficulties in struggling writers.

## 5. Limitations

Our results need to be interpreted with caution due to the limited sample size, and the fact that the research was embedded in a rather particular linguistic setting. Further studies should account for more types of handwriting assessment and possible differences that arise from/between them. Therefore, the present results should be further explored with a larger sample size of children in all grades, to be able to establish more accurate developmental and longitudinal markers to explore further the handwriting limitations of children at-risk. Moreover, future studies should consider.

## 6. Concluding remarks

Difficulties with handwriting can hinder the execution of other writing processes and in turn, impair overall text quality and proficiency (Berninger et al., 2008). Longer pauses within the letter could account for the fact that the writer may have not still fully integrated and automatized the symbolic-abstract shape of letters and, therefore, they have to either rely on visual feedback of the strokes (Alamargot et al., 2021). Alternatively, writers might need to scan through their sensori-motor integration graphical representations of the handwritten letters to be able to plan and reproduce the strokes sequentially (Longcamp et al., 2016). Further neuropsychological studies could help inform of any differences, delays, or divergences in the neuroanatomical integration and specificity of the handwritten automatization.

The present study has demonstrated that a 2,000 ms threshold may be adequate for exploring high-level writing processes (Schilperoord, 2002; Strömqvist et al., 2006; Alves et al., 2008), but it is not appropriate to understand transcription processes. A non-trivial proportion of schoolaged children, specially in the initial stages of learning to write and those with writing difficulties, have not yet automatized low-level writing processes and, therefore, pauses under 2,000 ms should be considered to account for the overall writing process. Overall, pauses amount to about half the time taken to compose a text (Strömqvist and Wengelin, 1999; Alamargot et al., 2007), and allow writers’ working memory to handle and access cognitive resources (McCutchen, 2000). If pauses due to handwritten activity can be pinpointed around the 500 ms threshold, future studies should aim to explore those pauses above 500 ms, to scrutinize thresholds for transcription activity, from accessing lexical information, to resolving spelling ambiguities (Wengelin, 2006; Brizan et al., 2015; Medimorec and Risko, 2017). The specific role of different types of pauses should be further explored both in text composition and process-specific tasks.

Letter features had a small to insignificant effect in children’s handwriting pauses. This finding in itself does not entirely resolve an instruction issue on which script to teach. Nonetheless, it indicates that choice of script does not substantially affect the cognitive load during handwriting. Teaching practices should be reassessed considering the real utility of carefully choosing a script type for the formal instruction of writing.

## Data availability statement

The datasets generated for this study are available on request to the corresponding author.

## Ethics statement

The studies involving human participants were reviewed and approved by Ethics Commitee of the Autonomous University of Barcelona. Written informed consent to participate in this study was provided by the participants’ legal guardian/next of kin.

## Author contributions

MP, NS, and OS contributed to the conception, design of the study, and wrote sections of the manuscript. MP built the database and wrote the first draft of the manuscript. NS performed the statistical analyses. All authors contributed to the article and approved the submitted version.

## Funding

This research was supported by Spanish grants 2015ACUP 00175 and PID2019-108791GA-I00, awarded to NS.

## Conflict of interest

The authors declare that the research was conducted in the absence of any commercial or financial relationships that could be construed as a potential conflict of interest.

## Publisher’s note

All claims expressed in this article are solely those of the authors and do not necessarily represent those of their affiliated organizations, or those of the publisher, the editors and the reviewers. Any product that may be evaluated in this article, or claim that may be made by its manufacturer, is not guaranteed or endorsed by the publisher.

## Footnotes

^1^Adaptation of the strokes per letter required for each letter-case of Soler-Vilageliu and Kandel (2012) can be found in Figure A1 of the Supplementary material.
