# Supplementary material for: In a split second: Handwriting pauses in typical and struggling writers
Source: Front Psychol. 2023 Jan 6;13:1052264. doi: 10.3389/fpsyg.2022.1052264 (PMC9853007; doi:10.3389/fpsyg.2022.1052264)
Supplement: Supplementary file 1 [file Table_1.DOCX]

Supplementary Material

| Figure A1. *Strokes by letter per type of script frequently used by children, based on Soler & Kandel, (2009).^a^* | | | | | |
| --- | --- | --- | --- | --- | --- |
| Cursive  Lower-case | No. of Strokes | Manuscript  Lower-case | No. of Strokes | Manuscript  Upper-case | No. of Strokes |
| a | 3 | a | 3 | A | 3 |
| b | 4 | b | 3 | B | 5 |
| c | 3 | c | 2 | C | 2 |
| d | 4 | d | 3 | D | 3 |
| e | 2 | e | 3 | E | 4 |
| f | 3 | f | 2 | F | 3 |
| g | 4 | g | 3 | G | 4 |
| h | 4 | h | 3 | H | 3 |
| i | 3 | i | 2 | I | 1 |
| j | 4 | j | 2 | J | 2 |
| k | 5 | k | 3 | K | 3 |
| l | 2 | l | 1 | L | 2 |
| m | 6 | m | 5 | M | 4 |
| n | 4 | n | 3 | N | 3 |
| o | 3 | o | 2 | O | 2 |
| p | 5 | p | 3 | P | 3 |
| q | 4 | q | 3 | Q | 3 |
| r | 3 | r | 2 | R | 4 |
| s | 3 | s | 3 | S | 3 |
| t | 3 | t | 2 | T | 2 |
| u | 4 | u | 2 | U | 2 |
| v | 4 | v | 2 | V | 2 |
| w | 5 | w | 4 | W | 4 |
| x | 4 | x | 2 | X | 2 |
| y | 4 | y | 2 | Y | 3 |
| z | 4 | z | 3 | Z | 3 |
| ^a^This classification of strokes per letter was firstly proposed by Meulenbroek and van Galen, (1990) based on the motor changes of direction needed to construct each letter. Font has been chosen as an illustrative way of representing the differences between case. Script MT Bold is the font that illustrates cursive, Courier New was chosen for script. | | | | | |
